# Zirconium Oxide as a Novel Material for Post-Endodontically Treated Teeth: Comparative Fracture Behavior of 3D-Printed Cobalt–Chromium, Milled Zirconium Oxide, and Quartz Fiber Posts

**DOI:** 10.3390/ma17225611

**Published:** 2024-11-17

**Authors:** Armin Sokolowski, Fernando Gustavo Warchomicka, Lukas Seebacher, Bernhard Remschmidt, Marcus Rieder, Lumnije Kqiku-Biblekaj, Alwin Sokolowski

**Affiliations:** 1 Division of Restorative Dentistry, Periodontology and Prosthodontics, Department of Dental Medicine and Oral Health, Medical University of Graz, 8010 Graz, Austria; armin.sokolowski@medunigraz.at (A.S.); lukas.seebacher@medunigraz.at (L.S.); lumnije.kqiku@medunigraz.at (L.K.-B.); 2 Institute of Materials Science, Joining and Forming, Graz University of Technology, 8010 Graz, Austria; fernando.warchomicka@tugraz.at; 3 Division of Oral and Maxillofacial Surgery, Department of Dental Medicine and Oral Health, Medical University of Graz, 8010 Graz, Austria; bernhard.remschmidt@medunigraz.at (B.R.); marcus.rieder@medunigraz.at (M.R.)

**Keywords:** zirconium oxide, post-endodontic treatment, fracture behavior, quartz fiber posts, root-treated teeth, cobalt–chromium

## Abstract

This study evaluates the mechanical properties of materials used in the post-endodontic restoration of root-treated teeth and examines their performance under stress to provide insights for material selection in clinical applications. Particular attention is given to zirconium oxide, which demonstrates promising characteristics due to its esthetic color and favorable material properties, positioning it as a potential material for future use in post-endodontic treatments. Three materials—prefabricated quartz fiber-reinforced composite, milled zirconium oxide, and additively manufactured cobalt–chromium—were evaluated using compressive-deflection tests. The specimens were subjected to a 45° compressive load extending 5 mm from a metal core. Their mechanical properties were analyzed using one-way ANOVA and Tukey’s post hoc test. Significant differences were observed among the materials. Quartz fiber posts, with the lowest force resistance, buckled at lower loads (143.3 ± 9.9 N), while zirconia posts failed in a brittle manner at higher forces (246.1 ± 97.2 N). Cobalt–chromium posts demonstrated the highest maximal force (323.2 ± 10.5 N, *p* < 0.001) and, unlike the other materials, bent rather than fractured. The failure patterns of the tested materials underscore the importance of careful material selection when restoring root-treated teeth. Despite displaying distinct fracture characteristics, zirconium oxide, due to its color, rigidity, and hardness, stands out as a promising material for future dental applications. Further research through randomized clinical trials is recommended to refine treatment approaches and optimize clinical outcomes.

## 1. Introduction

Modern post-endodontic materials, such as milled zirconia, provide esthetic benefits. However, compared to other state-of-the-art materials like selective laser-melted cobalt–chromium and glass fiber posts, milled zirconia displays unique properties and distinct fracture behaviors. Understanding these differences aids in making informed decisions when selecting posts for root-treated teeth.

The restoration of endodontically treated teeth often necessitates post core systems, especially when coronal tissue is insufficient for restoration. As an integral part of dental treatment options, post and core restorations hold significant importance in dental prosthetics [1]. They are essential in restoring the structural integrity and ensuring the long-term functionality of teeth, particularly in cases with significant substance loss. The longevity and fracture resistance of the treated teeth depend on the material chosen, the cementing method, and the post’s thickness [2,3]. While prefabricated systems, typically comprising quartz fiber or titanium posts, have been in use for some time, severely damaged teeth with minimal remaining tooth structure may require custom-made posts [4]. These are designed to distribute forces optimally, fit perfectly, and leverage the ferrule effect to minimize fracture risks, thus enhancing the tooth’s lifespan post-endodontically.

The success of post-endodontic tooth restoration is significantly influenced by the ferrule height and the number of remaining coronal walls [5,6]. These factors are key in reducing stress on the post and the remaining tooth tissue. Traditionally, custom-made cast post cores have been the method of choice for restoring teeth with extensive damage. However, such techniques can be time-consuming and require meticulous execution [7]. Cobalt–chromium alloys are widely recognized as one of the most prominent base metal alloys utilized in dentistry, demonstrating various successful clinical applications. They are characterized by their excellent biocompatibility, high strength, heat resistance, and non-magnetic properties, as well as their ability to resist wear, corrosion, and tarnish. The high modulus of elasticity of cobalt–chromium alloys provides the necessary strength and rigidity for reducing the weight of metal structures [8]. While most of the cobalt–chromium alloys used in the industrial and biomedical fields today originated from the research of Elwood Haynes during the previous century, the techniques used to process this material have since significantly changed [9]. With the appearance of selective laser melting (SLM), achieving predictable results in production, fitting, and material homogeneity has become more feasible. Consequently, this technology is increasingly becoming a part of routine dental restoration procedures [10]. Fiber-reinforced composite posts, introduced in the 1990s, incorporate materials like glass, quartz, or carbon fibers within an epoxy or methacrylate matrix. Their flexibility, dentin-like elasticity modulus, and metal-free composition make them ideal for esthetic applications, particularly in anterior teeth. However, clinical studies suggest a higher failure rate for these posts over ten-years, primarily due to breakage and loss of retention [11]. Contrarily, ceramic materials, particularly zirconium oxide, have been used extensively since 1969 due to their esthetic superiority over metals. Zirconium oxide’s high biocompatibility, resistance to corrosion, and chemical stability make it a preferred choice. Despite its brittleness and difficulty in removal or replacement when fractured, zirconium oxide posts demonstrate impressive success rates, with studies indicating a survival probability of over 80% over a decade [12]. The advancements in computer-assisted design and manufacturing (CAD/CAM) technology have revolutionized post and core restorations with ceramic materials, allowing the fabrication of accurately fitting, digitally designed restorations. It is beneficial from an esthetic perspective, where tooth-colored and white posts and cores are favored to avoid color discrepancies with adjacent teeth. Despite the prevalence of these materials, research into the fracture behavior of posts made from cobalt–chromium, zirconium oxide, and quartz fiber remains limited. While studies focus on the clinical performance and long-term success of post-restored teeth, post-fracture issues remain a significant complication in clinical practice. The comparative studies conducted by various researchers provide deeper insights into the performance and suitability of post materials. These studies demonstrate the superiority in mechanical strength of zirconium oxide posts, similar failure rates between fiber-reinforced composite posts and metal posts, and no significant differences in the success and survival rates of glass fiber posts compared with cast metal posts [13,14,15].

The hypothesis of this study is that there are no significant differences in the mechanical properties of the tested materials. The aim is to determine whether all materials demonstrate comparable characteristics suitable for the restoration of root canal-treated anterior teeth.

## 2. Materials and Methods

To compare the primary outcome measure with a power of 80% and a significance level of α = 5%, a sample size calculation determined that seven samples per group were needed to detect a difference of 10 percentage points between the groups, assuming a standard deviation of 6%. A 10% change in mechanical properties is considered a clinically meaningful difference. For this reason, ten samples per group were used to investigate the mechanical properties. Three different materials of post-endodontic posts were studied. Initially, ten quartz fiber-reinforced composite posts of ISO size four in green (X-Post, Dentsply Sirona), consisting of 60% quartz fibers and 40% epoxy resin, were reconstructed in the CAD software “Fusion 360 v.2.0.19994” (Autodesk, San Francisco, California, United States). The designs had identical post geometries and were reproduced using computer-aided manufacturing (CAM).

Ten zirconium oxide posts were milled from a zirconium oxide blank (Copran, Whitepeaks Dental Solutions, Hamminkeln, Germany) using a five-axis milling machine (CORiTEC 150i PRO, imes-icore). The milling process included a lateral support structure, which was later removed and polished.

The metal posts were additively manufactured from a biocompatible cobalt–chromium–tungsten powder (Remanium star CL; Dentaurum) under a nitrogen atmosphere, using a high-power fiber laser (Mlab cusing R; Concept Laser) with a laser power of 56 watts and a scanning speed of 500 mm/s. The layer thickness was set at 30 µm. After fabrication, the posts were sandblasted to achieve a homogenous surface.

A customized testing approach, as described by Lambjerg-Hansen et al. (1997), was selected over a standardized test for evaluating post-endodontically treated teeth because standardized tests generally lack the specificity needed to simulate the clinical environment of these restorations accurately [16]. Post-endodontic teeth have unique structural considerations and stress distributions, often involving weakened areas due to prior treatment. The customized method allows for the measurement of the stiffness, elastic limit, and fracture strength of posts under conditions that closely replicate clinical stresses. This tailored approach provides a more reliable assessment of post durability and stability, thus enhancing its clinical relevance [17].

**Figure 1:**
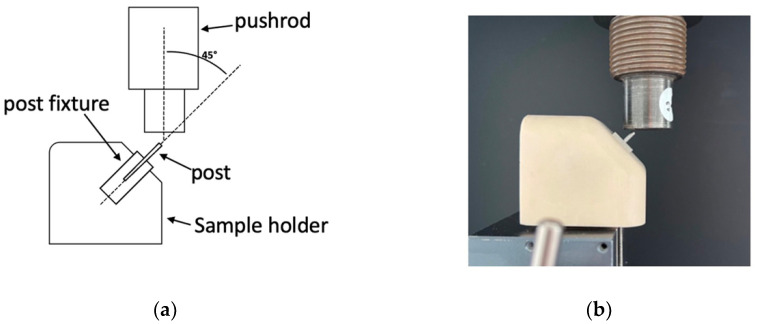
Testing machine with custom-made fixture clamping samples at 45° angle—schematic diagram (**a**), experimental setup (**b**).

Mechanical testing was carried out using a tensile testing machine with a maximum force range of 10 kN (Autograph AGS-X Series, Shimadzu, Kyōto, Japan). The sample extended 5 mm from a custom-made metal fixture and was clamped at an angle of 45° (Figure 1a) to the post axis in the testing machine (Figure 1b).

The sample holder, along with the samples, was aligned and fixed in the screw clamp of the testing machine and pre-set to ensure consistent testing conditions. This setup ensured that an angle of 45° between the post axis and the test body was reproducible for each test. A pushrod of 20 mm in diameter was subjected to a displacement rate of 2 mm/min until the fracture of the sample or maximum displacement of 2.5 mm first occurred. The force and displacement were recorded at 100 Hz. The statistical analysis compared the three materials’ stiffness, elasticity limit, and maximum force. A preload of 10 N was applied to ensure the specimen was fully settled in the fixture. This prevented any initial shifting or compliance effects, so that all recorded values accurately represent the material’s response.

The stiffness, elastic limit, and maximum force were derived from the force–displacement data obtained from mechanical testing. Stiffness was calculated as the ratio of the applied force to the corresponding displacement within the linear (elastic) region of the force–displacement curve. Specifically, the force at the elastic limit was divided by the maximum elastic displacement in mm at that point. This approach provided a measure of stiffness in N/mm, capturing the material’s resistance to elastic deformation. The elastic limit was identified as the point on the force–displacement curve where significant deviation from linearity first occurred, indicating the transition from elastic to plastic behavior. This method allowed us to objectively define the onset of plastic deformation without requiring an offset strain, focusing instead on the visual inflection point where elastic behavior ceased. To assess the load-bearing capacity of each material, the maximum applied force before failure was recorded. Due to the distinct failure behaviors—such as brittle for zirconium oxide, ductile in metal posts, and partial structural breakdown in quartz fibers—this maximum force was selected as a consistent indicator of material performance across varied material types.

The mean and standard deviation for each parameter were calculated and rounded to two decimal places for clarity. An ANOVA test was conducted to compare the three materials, with the significance level set at *p* < 0.05. A Tukey post hoc test was employed to find statistical differences between the materials. All calculations were performed using SPSS Statistics Version 27 (IBM, New York, NY, USA).

The fracture surface and material surfaces of the specimens were observed using a Field-Emission Gun scanning electron microscope (FEG-SEM), Tescan Mira 3 (Tescan, Brno, Czech Republic). Images were acquired at an acceleration voltage of 10 kV using a secondary electron detector. Non-conductive samples were coated with carbon (~10 nm thickness).

## 3. Results

**Figure 2:**
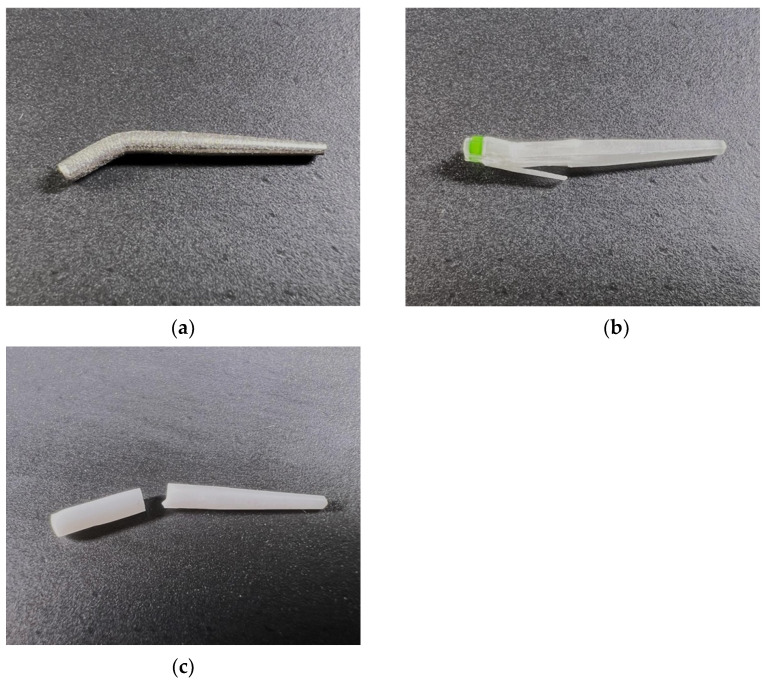
Samples (posts) after test at 45°: (**a**) cobalt–chromium alloy, (**b**) quartz fiber-reinforced composite, (**c**) zirconium oxide.

Tests at 45° showed distinct behaviors among the three tested post materials, as observed in Figure 2. The metal posts exhibited plastic deformation without rupture at the end of the test. Samples of zirconium oxide and quartz fiber groups resulted in a definable fracture. A consistent fracture pattern was observed across the two groups. The fracture line was almost horizontal, perpendicular to the post axis, with no splintering. The quartz fiber-reinforced composite posts bent at a defined maximum load (N), but the fragments remained connected due to undamaged fibers. The fracture line was identifiable by a cloudy-white discoloration, running vertically along the fibers.

**Figure 3:**
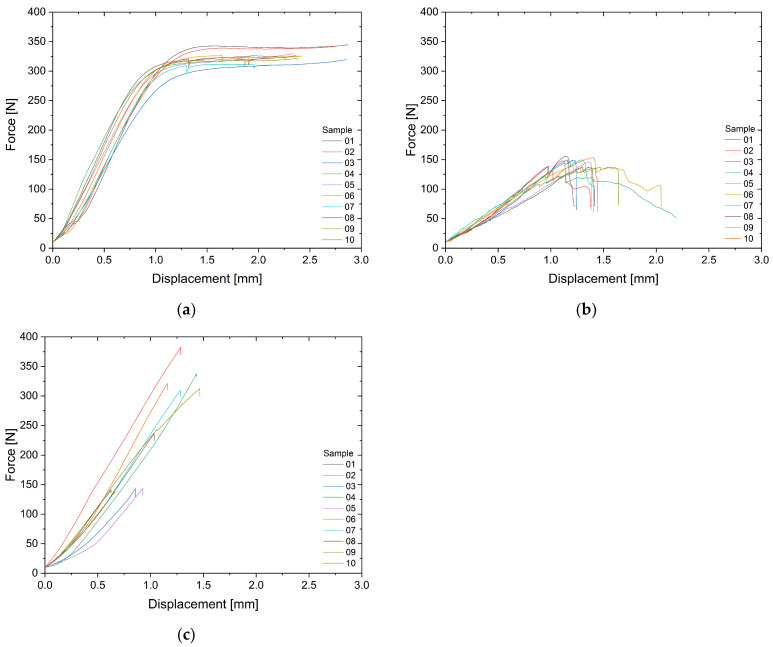
Force–displacement curves depicting group characteristics. (**a**) 10 cobalt–chromium posts, (**b)** 10 quartz fiber posts, (**c**) 10 zirconium oxide posts.

Figure 3 shows force–displacement curves for each group, with a representative curve provided for each material. Cobalt–chromium posts reached a plateau in the range of 300–350 N, indicating that these posts reached their maximum load limit without apparent fracture or cracking (Figure 3a). The force–displacement diagrams in Figure 3b revealed visible micro-cracks at low forces in quartz fiber posts. The transition to the plastic phase was not clear, with a prolonged displacement phase and repeated fiber cracking. The diagram in zirconium oxide posts showed a linear increase in force without plastic deformation with typical abrupt failure below 2 mm displacement (Figure 3c).

Table 1 summarizes the findings of the three materials. Zirconium oxide posts showed no plastic behavior, with high stiffness (224.5 ± 43.8 N/mm) and maximum force and an elastic limit of 246.1 ± 97.2 N, indicating a lack of ductility.

**Figure 4:**
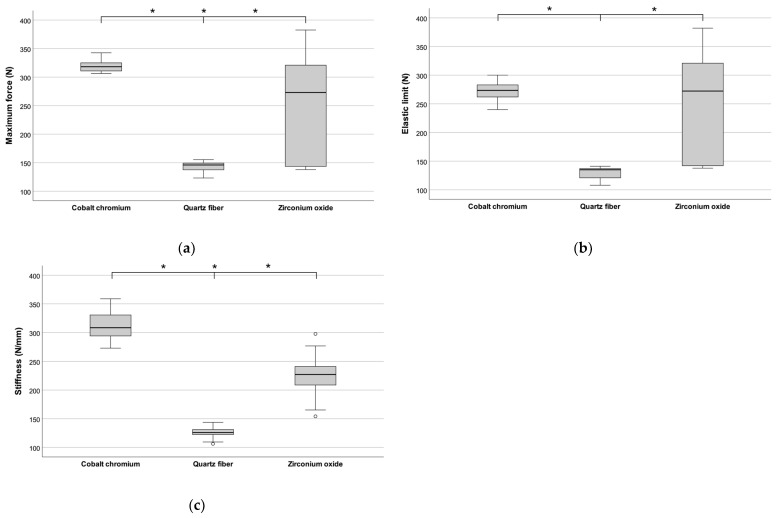
Box plots of tensile testing results. (**a**) Maximum force, (**b**) elastic limit, (**c**) stiffness. Statistically significant results marked with asterisk (*) (n = 10, *p* < 0.001).

Quartz fiber and cobalt–chromium posts demonstrated lower elastic limit values compared to their maximum force values, indicating some plasticity. Cobalt–chromium posts exhibited the highest maximum force (323.2 ± 10.5 N) without visible damage during testing. In contrast, quartz fiber posts showed the lowest values across all metrics: elastic limit (129.8 ± 11.0 N), maximum force (143.3 ± 9.9 N), and stiffness (162.1 ± 12.2 N/mm). Zirconium oxide posts displayed stiffness values more comparable to cobalt–chromium posts but performed differently under maximum force. A detailed statistical evaluation, including a Tukey post hoc test, is shown in Figure 4. Statistically significant differences were observed in the studied parameters across all three materials. Quartz fiber posts had minimal variation in stiffness values, concentrated between 106.2 N/mm and 143.6 N/mm. Zirconium oxide posts showed the widest range of stiffness values, from 154.2 N/mm to 297.6 N/mm (Table 1, Figure 4a).

Cobalt–chromium posts fell in between, with a stiffness range of 272.7 N to 358.9 N/mm. The elasticity limit of cobalt–chromium posts varied from 240 N to 300 N. Quartz fiber posts had a mean elastic limit of 129.8 N.

The cobalt–chromium posts exhibited the highest maximum force values, with low variation, ranging from 310.7 N to 342.3 N, with a mean value of 323.2 N. In contrast, the zirconium oxide posts showed the largest range, with maximum force values from 137.8 N to 382.1 N and a mean value of 246.1 N.

**Figure 5:**
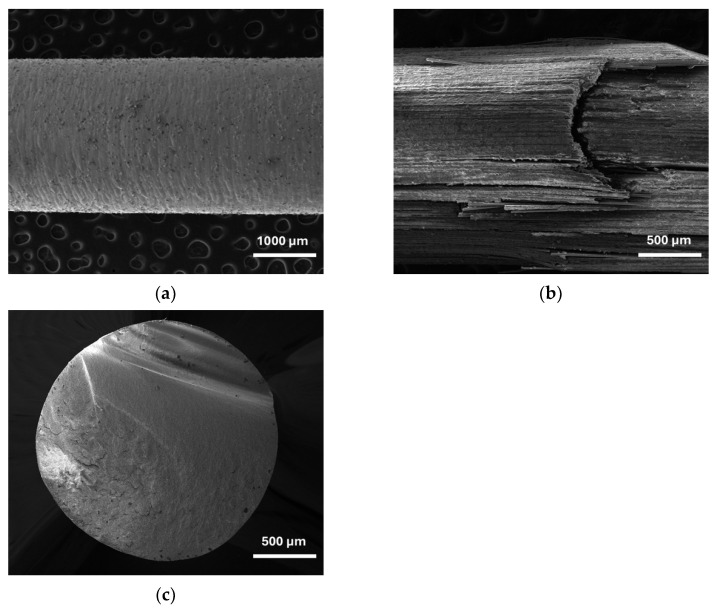
SEM images at the posts’ maximum displacement point: (**a**) Surface of cobalt–chromium alloy. (**b**) Detail of the fracture zone of the quartz fiber post. (**c**) Detail of the fracture surface of the zirconium oxide post.

Figure 5 shows the SEM images of the surfaces of the metal and quartz fiber posts, as well as the fracture surface of the zirconium oxide post. The additively manufactured cobalt–chromium alloy exhibited a crack-free surface with visible printing layers at maximum deflection (Figure 5a), while the quartz fiber posts exhibited mixed damage under the same testing conditions, with observable micro-cracks in the matrix, delamination/debonding, and fiber rupture (Figure 5b). These observations in the quartz fiber posts agree with comparable SEM investigations carried out in three-point tested samples [18]. The zirconium oxide posts demonstrated a clean fracture surface with a homogeneous material structure, free from any porosities (Figure 5c).

## 4. Discussion

The hypothesis of our study, positing no significant differences in the mechanical parameters of the tested materials, can be refuted. The statistical analysis clearly indicated significant variances among the materials, emphasizing the importance of material selection in post-endodontic treatments. Clinical relevance is paramount, especially considering the high occlusal loads that post-endodontic teeth must endure. The focus on compressive-deflection tests under a 45° angled load is particularly relevant, as this angle closely mimics the human interincisal angle and reflects the non-axial nature of masticatory forces in the anterior region. Zirconium oxide posts exhibited a high maximum force (246.1 ± 97.2 N) and stiffness; however, their brittleness was evident due to their limited plastic deformation. The large variance in tensile strength within the zirconium oxide posts group could likely be attributed to the manufacturing process. Manual interventions, such as the removal of lateral support structures during fabrication, may introduce micro-weaknesses, influencing the broad span of force.

Cobalt–chromium posts demonstrated a high maximum force (323.2 ± 10.5 N) without fracturing under pressure. Instead, they deformed, which can be attributed to their low yield strength. These findings confirm their established reliability and effectiveness in clinical settings. Quartz fiber posts, however, were notable for their relatively low maximum force (143.3 ± 9.9 N). The occurrence of micro-cracks at loads as low as 20 N suggests a propensity for fracturing (Figure 5c) under increased loads, even though they are capable of withstanding physiological masticatory forces in the anterior region. This study also shed light on the challenges in the manufacturing processes of these posts. While computer-assisted design and additive manufacturing (selective laser melting) showed efficiency and precision, subtractive manufacturing faced several limitations, particularly in the production of zirconium oxide posts. The issues encountered, such as tool vibrations and the need for manual post-processing, potentially contributed to the variability in the results and highlight the need for further enhancements in manufacturing techniques.

In clinical practice, cost is an important factor influencing material selection. While the initial investment for a cobalt–chromium laser melting device is high, the ongoing costs for cobalt–chromium powder and maintenance are relatively low. This cost structure enables amortization with higher production volumes, making cobalt–chromium posts cost-effective in high-demand settings. On the other hand, quartz fiber posts are industrially manufactured and comprise multiple components, which results in moderately higher costs due to the pricing set by manufacturing companies. Thus, these posts lack the customization potential of cobalt–chromium and zirconium oxide posts, manufactured in dental laboratories. Zirconium oxide posts can be produced using milling machines, with lower initial costs than laser melting devices. Zirconium oxide blanks exhibit high fracture resistance, while the operating costs remain low due to minimal tool wear, as the zirconia reaches its final hardness only after the sintering process.

Comparative studies further underscore the complexity of choosing the right material, highlighting that while some materials may exhibit higher strength, their failure modes and long-term performance under clinical conditions can differ significantly. One study conducted a notable in vitro fracture analysis using 40 extracted human single-rooted premolars [13]. These teeth were subjected to root canal treatments and then categorized into four groups, including one with Zirconium Oxide post core constructions. The testing, involving a 130° angle load until fracture, revealed that the zirconium oxide group exhibited significantly higher fracture resistance (315.4 ± 53.4 N) than all other groups. The lowest test values were observed in the cast metal post core group. Notably, the majority of fractures occurred in the cervical half of the root, rendering them irreparable. This study underscores the superior mechanical strength of zirconium oxide posts, albeit highlighting their vulnerability to catastrophic failure modes.

In another comprehensive investigation, Martins et al. conducted a detailed literature review and meta-analysis comparing fiber-reinforced composite posts and metal posts in endodontically treated teeth [14]. This study included ten research works involving 704 participants and 844 posts. The analysis covered aspects like absolute and relative failure, differentiating between anterior and posterior regions. The conclusion drawn was that there was no significant difference between the posts in terms of survival after failures. However, a limitation of this study was that only six of the included studies had an average evaluation period of more than five years, suggesting the need for longer-term evaluations to fully understand the implications of post material choice.

Another study conducted a fatigue testing study where all specimens were subjected to simultaneous mastication simulation and thermocycling. They found that both the cast metal and zirconia post-and-core groups exhibited similar fatigue resistance, surpassing that of the fiber-reinforced groups. A different approach was taken in another study with a randomized double-blind study, focusing on the success of glass fiber posts versus cast metal post [15]. The study involved human teeth treated endodontically without ferrule, comparing 72 cast metal posts and 111 glass fiber posts, all restored with metal–ceramic crowns. The five-year results indicated no significant differences in success and survival rates between the two materials. However, the study hinted at the necessity of long-term clinical studies to draw more definitive conclusions about long-term success.

These comparative studies, in conjunction with our findings, highlight the complex interplay of factors influencing the choice of post materials in endodontic treatments. While materials like zirconium oxide and cobalt–chromium show high tensile strength, their behavior under load and mode of failure differ significantly from materials like quartz fiber posts, which exhibit lower tensile strength but different failure characteristics. These differences are crucial for clinicians to consider, as the choice of post material can significantly impact the long-term success and functionality of endodontically treated teeth. The variation in study methodologies, angles of load application, and types of failure also demonstrate the need for a standardized approach in evaluating post materials to draw more universally applicable conclusions.

While this study offers valuable insights, a few minor limitations should be noted. The use of a non-standardized testing setup, including loading at a 45° angle rather than in a traditional bending configuration, was chosen to prioritize clinical relevance, though it limits direct comparability to standardized values. Additionally, the manual preparation of samples, particularly for zirconia, may have introduced variability due to potential effects of post-processing on material integrity. A preload of 10 N was necessary to stabilize the fixture, though it may slightly affect measurement precision at the lower end of the force range.

## 5. Conclusions

Based on the insights gained from this in vitro study, it can be concluded that the tested post materials can withstand the physiological forces encountered in the anterior region of the mouth. Metal posts, long regarded as the standard reference, exhibit clinically desirable mechanical properties but fall short in meeting esthetic requirements. Quartz fiber-reinforced composite posts present a transparent, simple, and cost-effective alternative. The low elastic modulus of the quartz fiber-reinforced composite helps to reduce stress peaks and the incidence of root fractures. However, they are more prone to loosening and damage due to fiber rupture and matrix cracking, as observed in the SEM images.

Modern esthetic post materials like zirconium oxide exhibit excellent mechanical properties. It is concluded that manufacturing identical posts from metals through additive manufacturing or from zirconium oxide through subtractive manufacturing is technically feasible. Moreover, these posts meet the clinical requirements in terms of mechanical properties. However, it is important to note that the workflow for the subtractive manufacturing of longer posts is not yet fully optimized and has room for improvement.

The findings of this study suggest that zirconium oxide could play a significant role in the future, potentially replacing metal-based custom-made posts in clinical practice. Its outstanding mechanical properties and biocompatible nature, as well as its white color, make it a promising candidate for use in dental restorations, especially in anterior regions. Future research should therefore focus more intensively on this material to fully explore its potential and limitations and develop optimal processing and application strategies.

To advance our understanding of post-and-core systems’ performance in clinical scenarios, randomized clinical trials incorporating various types of posts—both off-the-shelf and custom-made—are warranted. Such studies could offer valuable insights into the long-term success and durability of different post materials in real-world clinical settings.

## Figures and Tables

**Table 1:** The stiffness, elastic limit, and maximum force of the studied materials.

Parameter	Material	Min	Max	Mean	SD
Maximum force (N)	Cobalt–chromium	310.7	342.3	323.2	10.5
	Quartz fiber	122.3	156.2	143.3	9.9
	Zirconium oxide	137.8	382.1	246.1	97.2
Elastic Limit (N)	Cobalt–chromium	240.0	300.0	273.3	16.9
	Quartz fiber	108.0	141.0	129.8	11.0
	Zirconium oxide	137.8	382.1	246.1	97.2
Stiffness (N/mm)	Cobalt–chromium	272.7	358.9	313.2	28.5
	Quartz fiber	106.2	143.6	126.1	12.2
	Zirconium oxide	154.2	297.6	224.5	43.8

## Data Availability

The data supporting the findings of this study are available from the corresponding author upon reasonable request.
